# Supplementary material for: Oculocutaneous albinism variants in 28 consanguineous families and functional classification of a pathogenic deep intron variant in TYR
Source: Eur J Hum Genet. 2026 Mar 11;34(5):603–8. doi: 10.1038/s41431-026-02070-5 (PMC13171863; doi:10.1038/s41431-026-02070-5)
Supplement: Supplementary file 1 — Supplemental Figures S1-S2 [file 41431_2026_2070_MOESM1_ESM.pdf]

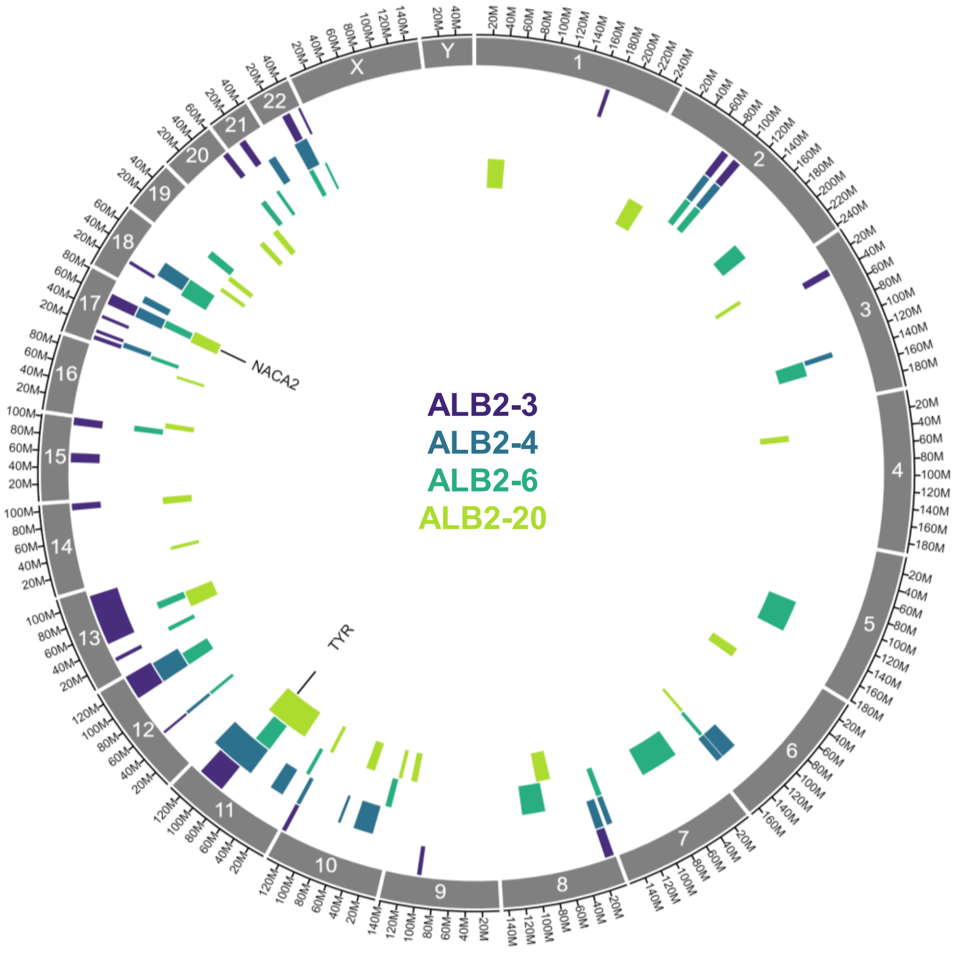

Fig. S1. Position of homozygosity-by-descent (HBD) regions in family ALB2. HBD regions from four affected family members are indicated with colour-codes. Only regions larger than 1 mbp is shown. Candidate genes in HBD regions, shared by all four affected individuals are indicated.

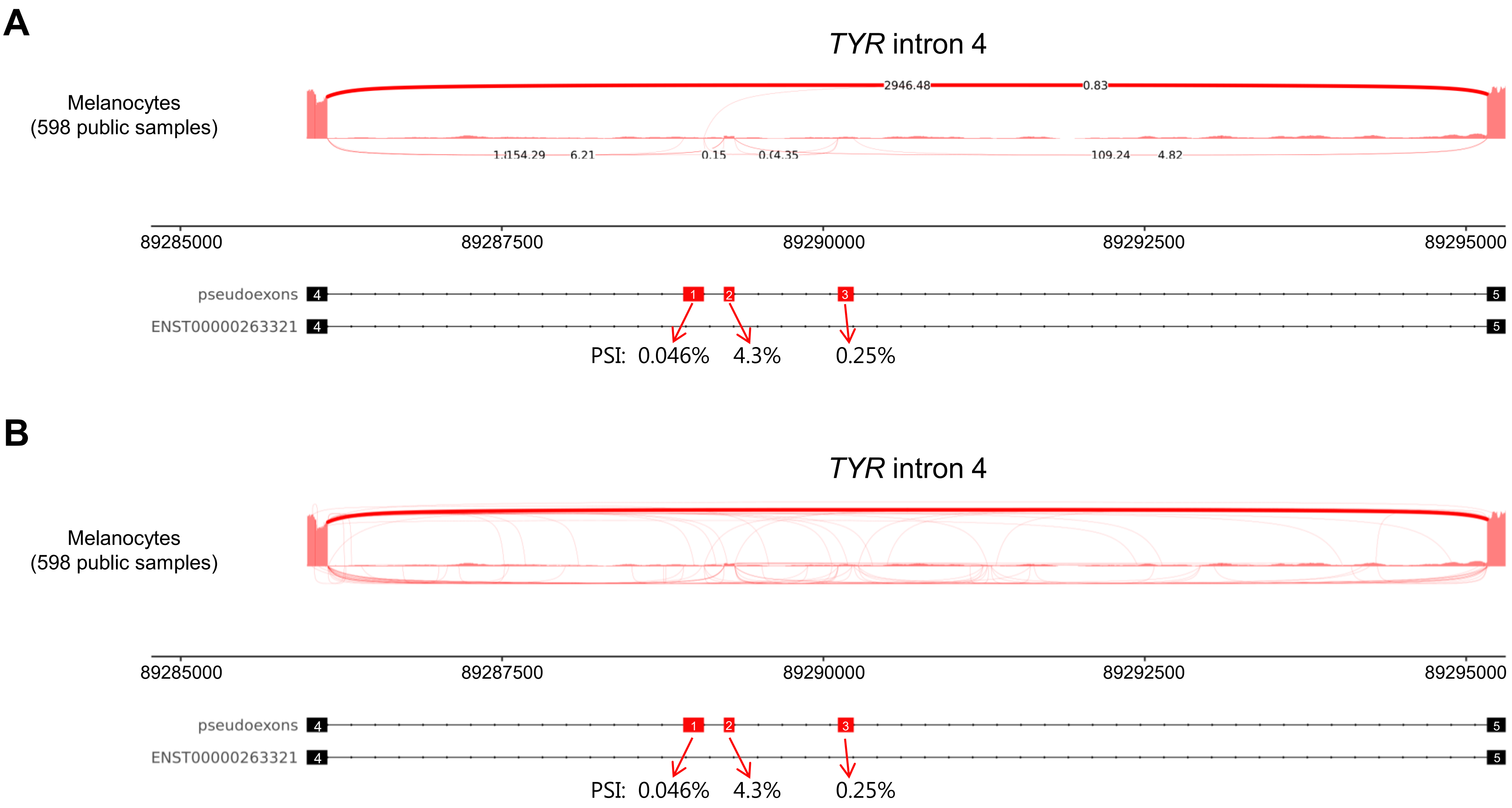

Fig. S2. Sashimi plots of intron 4 of the *TYR* gene. Plots show mean coverage and junction count based on 598 publicly available melanocyte RNA-seq datasets. The position of exon 4, exon 5 and pseudoexons PE1-3 are indicated below each plot. Exon-inclusion ratio in *TYR* mRNA (percent spliced in, PSI) is shown for each pseudoexon. In panel (**A**) only the junctions matching splicing between PE1-3 and normal exons are shown, in panel (**B**) all junctions in the region are indicated with line thickness correlated to mean junction count.
